# Expression of Concern: Governance and competitiveness evaluation of China’s financial asset management corporations

**DOI:** 10.1371/journal.pone.0338753

**Published:** 2025-12-15

**Authors:** The PLOS One Editors

The *PLOS One* Editors issue this Expression of Concern because this article [1] was identified as one of a series of submissions for which we have concerns about the peer review process. Readers are advised to interpret the article [1] with caution.

The Editors have no evidence of any author involvement in the peer review concerns.
