# Supplementary material for: Governance and competitiveness evaluation of China’s financial asset management corporations
Source: PLoS One. 2023 Dec 18;18(12):e0291695. doi: 10.1371/journal.pone.0291695 (PMC10727370; doi:10.1371/journal.pone.0291695)
Supplement: Attachment — Submitted filename: Response to Reviewers.docx [file pone.0291695.s002.docx]

**Response to Reviewers**

Dear external experts,

I am very grateful to PLoSONE for giving me the valuable opportunity to revise and improve my paper. I am also very grateful to the two experts for your helpful suggestions for my research. I have made improvements based on expert opinions and responded to this.

**Reviewer # 1 believes that**

1. There are some spelling errors in the initial draft of the paper. I carefully checked the paper and made careful spelling revisions. The meaning of "three boards and one layer" in Chinese is corporate governance structure, specifically referring to shareholders' meetings, board of directors, board of supervisors, and senior managers. I hereby replace it to eliminate the writing mistakes in Chinglish.

2. Revising the introduction based on expert opinions. The first paragraph of the introduction explains the research background and significance, the practical problems to be solved, and the goals to be achieved.

3. Updating references to highlight frontier and advanced academic researches. Such as:

Tarchouna A., Jarraya B., Bouri A. Do Board Characteristics and Ownership Structure Matter for Bank Non-Performing Loans? Empirical Evidence from US Commercial Banks[J]. Journal of Management and Governance, 2022, 26: 479–518. <https://doi.org/10.1007/s10997-020-09558-2>

[24]	Huang G., Ye F., Li Y. et al. Corporate Social Responsibility and Bank Credit Loans: Exploring the Moderating Effect of the Institutional Environment in China[J]. Asia Pacific Journal of Management, 2023, 40: 707–742. <https://doi.org/10.1007/s10490-021-09800-x>

Bouslah K., Hmaittane A., Kryzanowski, L. et al. CSR Structures: Evidence, Drivers, and Firm Value Implications[J]. Journal of Business Ethics, 2023, 185: 115–145. <https://doi.org/10.1007/s10551-022-05219-6>

Hopt K.J. Corporate Governance of Banks and Financial Institutions: Economic Theory, Supervisory Practice, Evidence and Policy [J]. European Business Organization Law Review, 2021, 22: 13–37. <https://doi.org/10.1007/s40804-020-00201-z>

4. The methodological contribution is clearly stated in the last part of the introduction. Based on internal and external governance mechanisms, a comprehensive evaluation index for enterprise competitiveness was constructed, and the factors and their degrees that affect the formation of competitiveness were studied by combining principal component analysis and grey relational analysis. This is a very innovative methodology.

5. Reviewers believe that a comparison should be made with past research to demonstrate the superiority and value of current research. The measurement of competitiveness is mostly based on countries or industries. I take China’s Financial Asset Management Corporation as the research object and construct a competitiveness evaluation index system based on corporate governance mechanism to measure China's AMC competitiveness in non-performing asset disposal market.

6. In the conclusion and Proposal, I added a discussion on the importance of measures and suggestions to highlight the theoretical and practical value of the research.

**Reviewer # 2 believes that**

1. Adding methodological content to the abstract of the paper. This paper proposes the theory of governance-performance-competitiveness for financial asset management corporations, and constructs a comprehensive competitiveness evaluation index system for financial asset management corporations based on internal and external governance mechanisms, and uses principal component analysis to measure and rank the competitiveness level of China’s financial asset management corporations, and establishes a grey correlation model to analyze the degree of correlation between competitiveness level and governance mechanisms. In addition, the paper explores the main layout of the non-performing asset disposal market through clustering and grouping.

2. The research sample for my paper is suitable and rich. There are currently 5 national financial asset management corporations and 59 local financial asset management corporations in China, but only 22 financial asset management corporations still publicly issue bonds in the market in 2021. According to Chinese financial law, if a financial enterprise publicly issues market bonds, it must disclose its main credit rating report or financial annual report to the society. Generally speaking, China’s corporations that are able to publicly disclose financial data are corporations with standardized operations, a certain scale, and status in their industry sector, which can largely reflect the true situation of this sector. Regarding the availability of financial data, I can only collect financial data from these 22 financial asset management corporations for research purposes. Regarding the selection of data for the 2021 financial annual report, this is because China's largest real estate company, Evergrande Group (www.evergrande.com), announced in 2021 that it was unable to fulfill its debt guarantee obligations, involving a debt of 2 trillion Chinese yuan. Local financial asset management corporations in China will therefore have good development opportunities, and each company is researching sustainable development strategies to enhance competitiveness. Therefore, selecting the financial data of 22 financial asset management corporations for 2021 as a sample is reasonable and appropriate, and can support the current research on financial asset management corporate governance in China.

3. Previous studies did not consider the dimensions of ESG (environmental, social, and governance) when constructing evaluation indicators for enterprise competitiveness. However, my study included ESG in the evaluation indicator system, and the ESG indicator used whether financial asset management corporations issued social responsibility reports as a proxy variable. From the operational practice of financial asset management corporations in China, the selection of this proxy variable is very appropriate, as only enterprises with more advanced asset management concepts will release social responsibility reports, which reflect the governance performance of financial asset management corporations.

4. Experts believe that the content of last part should be divided into two small parts: conclusions and measures. I added the main conclusion in the last section.

My research is very interesting and of great significance for the sustainable development of China’s financial asset management corporations and the understanding of the disposal entities in the China’s non-performing asset market. I am looking forward to my paper being published in PLoSONE. There are no disputes of interest or any academic misconduct in this paper. Please continue processing my manuscript with the editor. Thanks.

Regards,

Guangcan Huang

hgc@xupt.edu.cn
